# Cross-sectional and longitudinal determinants of serum sex hormone binding globulin (SHBG) in a cohort of community-dwelling men

**DOI:** 10.1371/journal.pone.0200078

**Published:** 2018-07-11

**Authors:** Prabin Gyawali, Sean A. Martin, Leonie K. Heilbronn, Andrew D. Vincent, Alicia J. Jenkins, Andrzej S. Januszewski, Anne W. Taylor, Robert J. T. Adams, Peter D. O’Loughlin, Gary A. Wittert

**Affiliations:** 1 School of Medicine, University of Adelaide, Adelaide, South Australia, Australia; 2 Freemasons Foundation Centre for Men’s Health, University of Adelaide, Adelaide, South Australia, Australia; 3 South Australian Health and Medical Research Institute (SAHMRI), Adelaide, South Australia, Australia; 4 NHMRC Clinical Trials Centre, University of Sydney, Camperdown, New South Wales, Australia; 5 Population Research and Outcomes Studies, University of Adelaide, Adelaide, South Australia, Australia; 6 The Health Observatory, University of Adelaide, Queen Elizabeth Hospital, Woodville, South Australia, Australia; 7 Chemical Pathology, SA Pathology, Adelaide, South Australia, Australia; University Hospital Center of Rennes, FRANCE

## Abstract

Despite its widespread clinical use, there is little data available from population-based studies on the determinants of serum sex hormone binding globulin (SHBG). We aimed to examine multifactorial determinants of circulating SHBG levels in community-dwelling men. Study participants comprised randomly selected 35–80 y.o. men (n = 2563) prospectively-followed for 5 years (n = 2038) in the Men Androgen Inflammation Lifestyle Environment and Stress (MAILES) study. After excluding men with illness or medications known to affect SHBG (n = 172), data from 1786 men were available at baseline, and 1476 at follow-up. The relationship between baseline body composition (DXA), serum glucose, insulin, triglycerides, thyroxine (fT4), sex steroids (total testosterone (TT), oestradiol (E2)), and pro-inflammatory cytokines and serum SHBG level at both baseline & follow-up was determined by linear and penalized logistic regression models adjusting for age, lifestyle & demographic, body composition, metabolic, and hormonal factors. Restricted cubic spline analyses was also conducted to capture possible non-linear relationships. At baseline there were positive cross-sectional associations between age (β = 0.409, p<0.001), TT (β = 0.560, p<0.001), fT4 (β = 0.067, p = 0.019) and SHBG, and negative associations between triglycerides (β = -0.112, p<0.001), abdominal fat mass (β = -0.068, p = 0.032) and E2 (β = -0.058, p = 0.050) and SHBG. In longitudinal analysis the positive determinants of SHBG at 4.9 years were age (β = 0.406, p = <0.001), TT (β = 0.461, p = <0.001), and fT4 (β = 0.040, p = 0.034) and negative determinants were triglycerides (β = -0.065, p = 0.027) and abdominal fat mass (β = -0.078, p = 0.032). Taken together these data suggest low SHBG is a marker of abdominal obesity and increased serum triglycerides, conditions which are known to have been associated with low testosterone and low T4.

## Introduction

Sex hormone binding globulin (SHBG) is a circulating homodimeric glycoprotein, primarily synthesised in the liver, that binds circulating sex steroids with high affinity [1, 2]. Variations in circulating SHBG levels are observed in a number of physiologic and pathological conditions. In men, low serum SHBG levels are associated with insulin resistance [3], obesity [4], non-alcoholic fatty liver disease (NAFLD) [5], type 2 diabetes (T2D) [6, 7], and cardiovascular disease [7]. Higher circulating SHBG is protective against the development of T2D in humans [8]. Overexpression of SHBG protects against T2D development in transgenic mice [9].

The synthesis and secretion of SHBG is subject to regulation by hormonal, metabolic, and nutritional factors [7, 10–13]. Factors shown to be inversely associated with SHBG levels include growth hormone [14], oestrogen (oestradiol) [15, 16], insulin [15, 17], body composition [15, 18, 19], intrahepatic fat [8, 18, 20], triglycerides [21, 22], monosaccharides [23], C-reactive protein (CRP) [24], and moderate alcohol consumption [25]. SHBG has been positively associated with testosterone [11, 15], follicle stimulating hormone [11], serum thyroxine [15], adiponectin [26], olive oil [27], red wine (resveratrol) [28], increasing age [29], physical activity [25] and resistance training [20, 30]. However, these data are derived largely from cross-sectional studies with a variety of limitations including small sample size, non-representative samples, restricted age ranges, limited biochemistry, non-concurrent variables [12, 15, 18, 22, 31] generally leading to inconclusive and inconsistent findings. Recent longitudinal data from the Boston Area Community Health/Bone Survey examining changes in anthropometric measures and sex steroids demonstrated that SHBG at baseline was not associated with changes in any of the included measures of body composition [32]. Another recent study (n = 1316) [33] undertook a secondary analysis of serum SHBG determinants. Their data suggested a possible effect of BMI, glucose, and lipids on SHBG levels. However, the follow-up period was relatively short, men were all older, and adjustment for confounders was limited [33].

Accordingly, in a longitudinal cohort study we examined cross-sectional and longitudinal relationships between lifestyle & demographic factors, body composition, metabolic, hormonal factors, and serum SHBG, simultaneously, in a large, community dwelling, representative cohort, of middle-aged to elderly Australian men.

## Subjects and methods

### Study design and participants

Participants for the present study were from the Men Androgen Inflammation Lifestyle Environment and Stress (MAILES) study. The MAILES study is a cohort of men aged 35 years and older, pooled from two existing population-based studies using identical sampling methods in the Northern and Western suburbs of Adelaide, South Australia: The Florey Adelaide Male Ageing Study (FAMAS) and North West Adelaide Health Study (NWAHS). A more detailed description of the study design, procedures, and recruitment was published previously [34]. Briefly, the FAMAS includes 1195 randomly-selected men, aged 35–80 years at recruitment who attended baseline clinic visits in 2002–2005 and follow-up clinic visits in 2007–2010. The NWAHS includes men and women aged 18-years at recruitment in 1999–2000, who attended three clinic waves (n = 2336). For the MAILES study, all FAMAS men and NWAHS age-matched (35–80 years at stage 2) men were included, yielding a final sample size of 2563 men at MAILES stage 1 (i.e., FAMAS baseline and NWAHS stage 2) and 2038 men at MAILES stage 2 (FAMAS 5-year follow-up and NWAHS stage 3). The mean follow-up period for the MAILES study is 4.9 years. The study was approved by the research ethics committees of the Royal Adelaide Hospital and the North Western Adelaide Health Service. A written informed consent form was given to the eligible participants who signed in-clinic.

**Fig 1:**
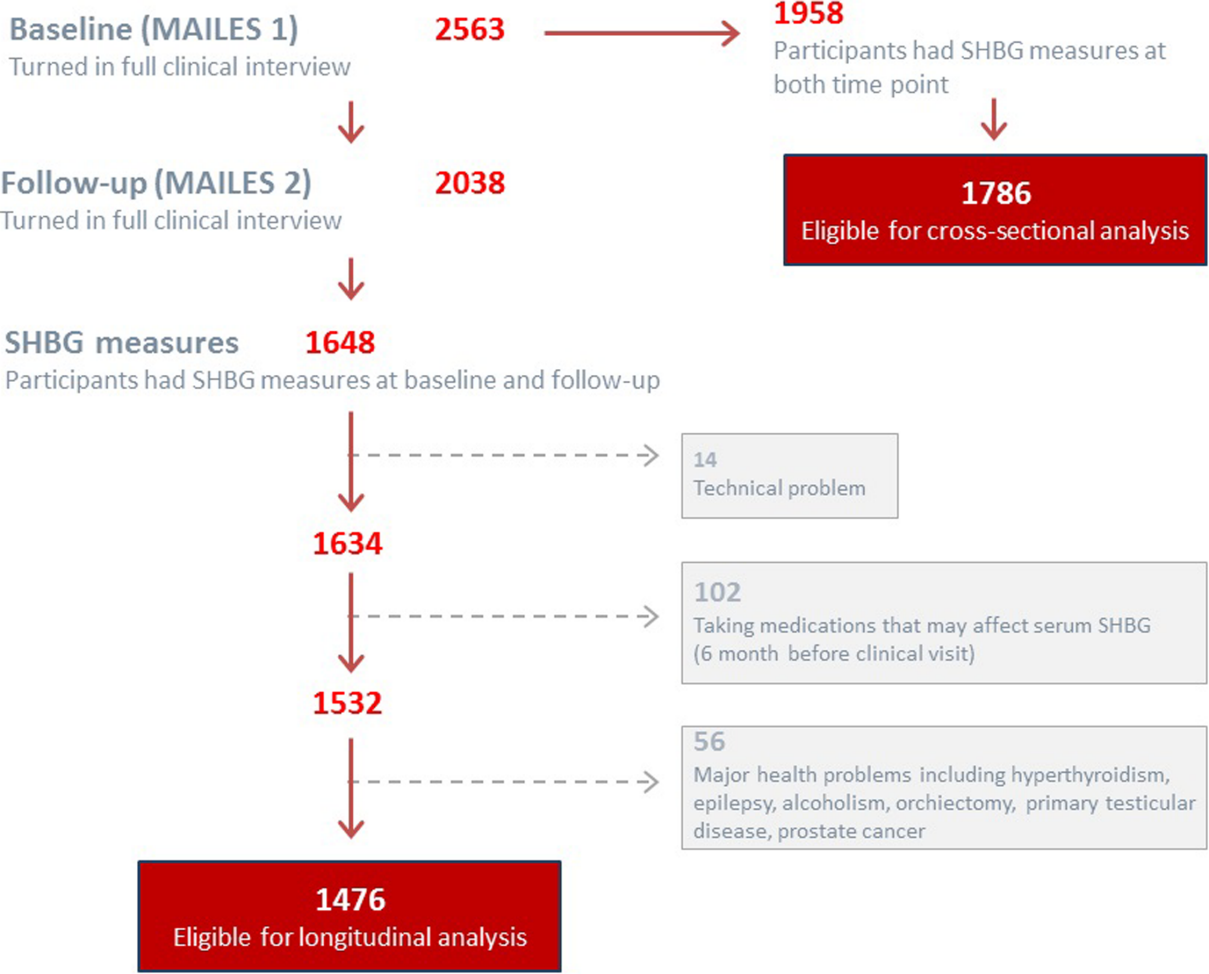
Description of MAILES sample enrolment. MAILES, men androgen inflammation lifestyle environment and stress; SHBG, sex hormone binding globulin.

For the current analysis, we excluded men with haemolysed samples (n = 14), confounding health conditions ((thyrotoxicosis, hypothyroidism, epilepsy, alcoholism, hepatitis c, heart bypass surgery, orchiectomy, primary testicular disease, & prostate cancer) (n = 56)) or on medications known to affect the hypothalamic pituitary gonadal (HPG) axis and hepatic SHBG synthesis (testosterone, antiandrogens, glucocorticoids, anti-epileptics, aromatase inhibitors, and thyroid hormone and anti-thyroid agents; n = 102), leaving a total sample of 1786 at baseline and 1476 at follow-up for analysis (Fig 1).

### Measurements

#### Socio-demographic and behavioural characteristics

Information on sociodemographic and behavioural characteristics (age, gender, physical activity, smoking status, and alcohol consumption), as well as medical history, including information about medical procedures, and medication use was obtained by a validated, self-report questionnaire [34]. Body composition was assessed using Dual-energy X-ray Absorptiometry (DXA) using the Lunar DPX+ and Prodigy pencil beam densitometers (Lunar Radiation Corporation, Madison, USA). Both densitometers show excellent congruence, with <1% difference in measurement across a range of measured areas [35].

#### Biochemical and hormonal assays

Full details of the laboratory methods and quality control data have been reported previously [34]. Briefly, blood samples were drawn between 8:00 AM and 11:00 AM after a 12-hour overnight fast. Samples were immediately placed on ice and transported to a laboratory certified by the National Association of Testing Authorities (NATA) within 4-hours, then centrifuged, fractionated, and serum stored at -80°C until measurement (between 1–13 months after collection). Samples were randomly ordered for assay and the laboratory technicians were blinded to participant characteristics. Serum SHBG levels were measured separately at baseline and follow-up by diluting serum to 1:21 through SHBG sample diluent, and then assayed using the Immulite Autoanalyser and a solid-phase, two-site chemiluminescent immunoassay (Siemens Medical Solutions, New York, USA; inter-assay CV: 4.0% at 32.3 nmol/L; lower detection limit: 0.17 nmol/L). Other plasma/serum measures included metabolic markers (glucose, triglycerides, insulin and alanine transaminase (ALT) activity), hormones (free thyroxine (fT4), total testosterone (TT) and oestradiol (E2)) and inflammatory markers (interleukin-6 (IL-6); tumour necrosis factor-alpha (TNF-α)); a marker of macrophage activation (myeloperoxidase activity (MPO)); and a marker of vascular endothelial inflammation (sE-selectin (eSel)). Inter-assay coefficient of variation (CV) were <10.6% for all measurements. Concentrations of all selected analytes are stable after multiple freeze-thaw cycles [36].

### Statistical analysis

Descriptive analyses of selected independents and outcome measures were conducted using chi-square- (categorical) and t-tests (continuous). To estimate cross-sectional and longitudinal determinants of SHBG variability, we implemented unadjusted, then age-adjusted and multi-adjusted linear regression and penalized logistic regression (using the least absolute shrinkage and selection operator (LASSO)) models. The cross-sectional models utilised serum SHBG levels and selected independents variables from baseline clinic visits. The longitudinal models fitted serum SHBG at follow-up (median 4.94 years follow-up, interquartile range 4.34–5.00) against selected determinants from baseline clinic visits. In separate models, we also assessed the effect of absolute changes between visits for SHBG determinants against the change in serum SHBG between visits. Normality and linearity assumptions were examined for all independents. Non-normal independents were log-transformed for analysis with results back-transformed for data presentation. Interaction effects were also examined for selected independents with resultant terms included in multi-adjusted models, where appropriate. All analyses were performed with the IBM SPSS statistical package (version 23.0 Armonk, NY, USA). A p-value < 0.05 was considered statistically significant. Explained variance (R^2^) of the models are also presented.

To access a possible non-linear relationships, restricted cubic splines were performed using STATA (version 15.0, STATA Corporation, Texas, USA) with 95% Confidence Intervals. Analyses were multi-adjusted with 3 knots at the 10th, 50th, and 90th percentiles of the distribution. To further explore these non-linear associations, tests for nonlinearity comparing a model with only the linear term to a model with the linear and restricted cubic spline terms were conducted using likelihood ratio tests (alternate middle knot locations). If a test for nonlinearity was not significant, a test for linearity was conducted comparing a model with the linear term to a model with only the covariates of interest (S1A–S1H Fig).

## Results

There were 1786 men at baseline, with a mean age of 55 years. At follow-up, complete data were available for 1476 men with a mean age 59 years. The characteristics of the MAILES stage 1 (baseline) and MAILES stage 2 (follow-up over 4.9 years) study populations are summarised in Table 1. No significant difference exists between baseline characteristics of those subjects who missed the follow-ups (n = 310) and those who did not (data have not been shown).

**Table 1:** Characteristics of the study participant at baseline and 5- year follow-up.

Parameters	Baseline (n = 1786)	5-Year follow-up (n = 1476)	P-value
Age,Years	55.0±12.2	59.0±11.5	**<0.001**
BMI (Kg/m^2^)	28.5±4.5	28.8±4.5	**<0.001**
Abdominal total fat mass (%)	34.8±8.2	36.3±8.0	**<0.001**
Triglycerides (mmol/L)*	1.79±1.42	1.73±1.33	0.157
Glucose (mmol/L)	5.20±1.41	5.49±1.42	**<0.001**
Insulin (μIU/mL)^a^	10.9±9.3	9.43±8.81	**<0.001**
ALT activity (U/L)^b^	33.8±19.1	32.0±18.4	**0.005**
fT4 (pmol/L)	14.4±2.3	16.2±2.81	**<0.001**
TT (nmol/L)	17.0±5.8	16.4±5.7	**<0.001**
E2 (pmol/L)^c^	96.3±42.8	94.6±35.2	**<0.001**
SHBG (nmol/L)	33.0±13.5	33.5±13.6	**<0.001**
IL-6 (pg/mL)	2.03±1.76	2.07±1.98	0.331
TNF-α (pg/mL)	1.94±2.88	2.07±2.67	0.064
MPO activity (μg/L)	208.4±295.6	181.7±199.8	**0.002**
eSel (ng/mL)	36.8±16.9	36.4±17.2	0.461

Values are mean ± standard deviation, unless stated otherwise.

Statistically significant associations (P <0.05) are shown in bold.

BMI, body mass index; ALT, alanine transaminases; f T4, free thyroxine; TT, total testosterone; E2, oestradiol; SHBG, sex hormone binding globulin; IL-6, interleukin 6; TNF-α, tumour necrosis factor alpha; MPO, myeloperoxidase; eSel, sE-selectin.

* geometric mean was 1.467 & median was 1.433 at baseline and mean was 1.493 & median was 1.455 at 5 year follow-up respectively.

^a^ n = 1099.

^b^ n = 1096.

^c^ n = 1136.

The cross-sectional analysis at MAILES stage 1 is shown in Table 2. In unadjusted analysis, SHBG was positively associated with age, fT4, TT and E2, and inversely associated with alcohol consumption, smoking, abdominal total fat mass, triglycerides, glucose, insulin, ALT activity, and eSel levels. After adjustment for age, SHBG remained positively associated with fT4, TT and E2 and inversely associated with smoking, abdominal total fat mass, triglycerides, glucose, insulin, ALT activity, IL-6, MPO activity and eSel levels. After multi-adjustment SHBG was positively associated with age, fT4 and TT and inversely associated with abdominal fat mass, triglycerides and E2. LASSO procedure selected TT, age, and f T4 respectively for having the highest positive regression coefficient while triglycerides and abdominal fat mass respectively for having the highest negative regression coefficient.

**Table 2:** Unadjusted, age-adjusted and multi-adjusted generalized linear regression and lasso regression model to estimate cross-sectional determinants of SHBG in community dwelling men (n = 1786).

Determinants/factors	Unadjusted model			Age-adjusted			Multi-adjusted (Full model)		LASSO regression	
	Standardized β	P-value	R^2^	Standardized β	P-value	R^2^	Standardized β	P-value	Standardized β	P-value
**Demographic, behavioural & anthropometric factors**										
Age, Years	**0.355**	**<0.001**	**0.126**	-	-	-	**0.409**	**<0.001**	**0.418**	**<0.001**
Physical activity	0.022	0.375	0.000	0.008	0.724	0.132	-0.012	0.667		
Alcohol consumption	**-0.088**	**<0.001**	**0.008**	-0.044	0.055	0.127	-0.041	0.145		
Smoking status	**-0.078**	**0.001**	**0.006**	**-0.149**	**<0.001**	**0.148**	0.005	0.873		
Abdominal total fat mass (%)	**-0.233**	**<0.001**	**0.054**	**-0.302**	**<0.001**	**0.202**	**-0.068**	**0.032**	**-0.081**	**0.006**
**Blood chemistry & hormones**										
Triglycerides (mmol/L)	**-0.251**	**<0.001**	**0.063**	**-0.227**	**<0.001**	**0.177**	**-0.112**	**<0.001**	**-0.121**	**<0.001**
Glucose (mmol/L)	**-0.061**	**0.010**	**0.004**	**-0.126**	**<0.001**	**0.141**	0.033	0.269		
Insulin (μIU/mL)^a^	**-0.171**	**<0.001**	**0.029**	**-0.201**	**<0.001**	**0.182**	0.002	0.945		
ALT activity (U/L)^b^	**-0.187**	**<0.001**	**0.035**	**-0.108**	**<0.001**	**0.152**	-0.029	0.350		
fT4 (pmol/L)	**0.105**	**<0.001**	**0.011**	**0.120**	**<0.001**	**0.139**	**0.067**	**0.019**	**0.086**	**0.002**
TT (nmol/L)	**0.535**	**<0.001**	**0.286**	**0.581**	**<0.001**	**0.461**	**0.560**	**<0.001**	**0.539**	**<0.001**
E2 (pmol/L)^c^	**0.110**	**<0.001**	**0.012**	**0.084**	**0.002**	**0.141**	**-0.058**	**0.050**		
**Inflammatory markers**										
IL-6 (pg/mL)	-0.017	0.514	0.000	**-0.065**	**0.008**	**0.123**	-0.007	0.795		
TNF-α (pg/mL)	-0.003	0.896	0.000	-0.023	0.356	0.117	-0.019	0.504		
MPO activity (μg/L)	-0.042	0.111	0.002	**-0.024**	**0.001**	**0.117**	0.011	0.705		
eSel (ng/mL)	**-0.178**	**<0.001**	**0.032**	**-0.160**	**<0.001**	**0.143**	-0.014	0.651		

Statistically significant associations (P <0.05) are shown in bold. Multi-adjusted generalized linear model R^2^ was 0.537 and lasso regression model R^2^ was 0.530. ALT,alanine transaminases; fT4,free thyroxine; TT,total testosterone; E2,oestradiol; SHBG,sex hormone binding globulin; IL-6,interleukin 6; TNF-α, tumour necrosis factor alpha; MPO,myeloperoxidase; eSel,sE-selectin.

^a^ n = 1097.

^b^ n = 1095.

^c^ n = 1134.

The regression estimates of longitudinal analysis are shown in Table 3. Unadjusted analysis of the longitudinal data showed significant a positive association between absolute SHBG at 4.9 years and age, physical activity, fT4, TT, E2, Il-6, and TNF-α at baseline and inverse association with alcohol consumption, abdominal total fat mass, triglycerides, glucose, insulin, ALT activity, MPO activity, and eSel levels at baseline with absolute SHBG levels at 4.9 years. After adjustment for age, SHBG remained positively associated with fT4, TT and TNF-α and inversely with alcohol consumption, abdominal total fat mass, triglycerides, glucose, insulin, ALT activity, MPO activity and eSel levels. After multi—adjustment SHBG was positively associated with age, fT4 and TT and inversely associated with abdominal total fat mass and triglycerides. LASSO procedure selected TT, age, and fT4 respectively for having highest positive regression coefficient while triglycerides and abdominal fat mass for having highest negative regression coefficient.

**Table 3:** Unadjusted, age-adjusted and multi-adjusted generalized linear regression and lasso regression model to estimate longitudinal determinants of SHBG in community dwelling men (n = 1476).

Determinants/factors	Unadjusted model			Age-adjusted			Multi-adjusted (Full model)		LASSO regression	
	Standardized β	P-value	R^2^	Standardized β	P-value	R^2^	Standardized β	P-value	Standardized β	P-value
**Demographic, behavioural & anthropometric factors**										
Age, Years	**0.320**	**<0.001**	**0.103**	-	-	-	**0.406**	**<0.001**	**0.419**	**<0.001**
Physical activity	**0.060**	**0.025**	**0.004**	0.045	0.075	0.114	0.005	0.874		
Alcohol consumption	**-0.106**	**<0.001**	**0.011**	**-0.070**	**0.006**	**0.110**	-0.056	0.083		
Smoking status	0.006	0.827	0.000	**-0.059**	**0.018**	**0.107**	0.050	0.135		
Abdominal total fat mass (%)	**-0.243**	**<0.001**	**0.059**	**-0.292**	**<0.001**	**0.143**	**-0.078**	**0.032**	**-0.081**	**0.014**
**Blood chemistry & hormones**										
Triglycerides (mmol/L)	**-0.224**	**<0.001**	**0.050**	**-0.208**	**<0.001**	**0.146**	**-0.065**	**0.027**	**-0.088**	**0.006**
Glucose (mmol/L)	**-0.094**	**<0.001**	**0.009**	**-0.162**	**<0.001**	**0.129**	-0.049	0.145		
Insulin (μIU/mL)^a^	**-0.157**	**<0.001**	**0.025**	**-0.178**	**<0.001**	**0.132**	-0.009	0.806		
ALT activity (U/L)^b^	**-0.239**	**<0.001**	**0.057**	**-0.168**	**<0.001**	**0.158**	-0.054	0.118		
fT4 (pmol/L)	**0.057**	**0.030**	**0.003**	**0.079**	**0.001**	**0.109**	**0.040**	**0.034**	**0.038**	**0.011**
TT (nmol/L)	**0.450**	**<0.001**	**0.202**	**0.489**	**<0.001**	**0.339**	**0.461**	**<0.001**	**0.448**	**<0.001**
E2 (pmol/L)^c^	**0.098**	**0.004**	**0.010**	0.047	0.150	0.129	-0.023	0.490		
**Inflammatory markers**										
IL-6 (pg/mL)	**0.062**	**0.016**	**0.004**	0.018	0.470	0.104	0.047	0.150		
TNF-α (pg/mL)	**0.074**	**0.005**	**0.006**	**0.056**	**0.025**	**0.109**	0.037	0.243		
MPO activity (μg/L)	**-0.093**	**<0.001**	**0.009**	**-0.076**	**0.002**	**0.110**	0.037	0.253		
eSel (ng/mL)	**-0.169**	**<0.001**	**0.029**	**-0.151**	**<0.001**	**0.128**	0.045	0.208		

Statistically significant associations (P < 0.05) are shown in bold. Multi-adjusted generalized linear model R^2^ was 0.420 and lasso regression model R^2^ was 0.404. ALT,alanine transaminases; fT4,free thyroxine; TT,total,testosterone; E2,oestradiol; SHBG, sex hormone binding globulin; IL-6,interleukin 6; TNF-α, tumour necrosis factor alpha; MPO activity, myeloperoxidase; eSel,sE-selectin.

^a^ n = 748.

^b^ n = 744.

^c^ n = 789.

To account for the effect of changes in selected independent variables on the outcome measure, we modelled the absolute difference for independents between clinic visits against the change in SHBG at follow-up (S1 Table). Furthermore, possible non-linear relationships between SHBG levels and independents have been assessed with multi-adjusted restricted cubic splines with 3 knots at the 10th, 50th, and 90th percentiles of the distribution. We found evidence of non-linear associations between SHBG levels and triglycerides (*P*_non-linearity_ < 0.001)), glucose (*P*_non-linearity_ < 0.001), insulin (*P*_non-linearity_ < 0.001), alanine transaminases (*P*_non-linearity_ < 0.001), oestradiol (*P*_non-linearity_ < 0.0001), IL-6 (*P*_non-linearity_ < 0.001), TNF-α (*P*_non-linearity_ < 0.001) and eSel (*P*_non-linearity_ < 0.001) (S1A–S1H Fig). To further explore these non-linear associations, we applied generalized models, with alternate middle knot locations specified by visual inspection and likelihood ratio tests of the corresponding regression curves (S1A–S1H Fig). With the exception of E2 and ALT activity all other independent variables within and above the cut-off were similar to original linear regression model with continuous variables (S2 Table).

## Discussion

This study showed a positive relationship of serum SHBG levels with age, thyroxine and total testosterone, and an inverse relationship with abdominal total fat mass, triglycerides and oestradiol at baseline. Longitudinally, there was a positive relationship of SHBG levels with baseline age, thyroxine, and total testosterone and an inverse relationship with abdominal total fat mass, and triglycerides.

Our finding of a positive association between age and SHBG is consistent with most previous studies [29, 37–39]. The only study to find no association between SHBG levels and age included participants younger than 45 years [15]. We also did not find any significant association of serum SHBG levels and age among men younger than 45 years (n = 458) (data not shown).

An independent inverse association between SHBG and obesity as defined by body mass index (BMI) and waist circumference (WC) has been reported cross-sectionally [29, 40, 41] and longitudinally [19, 22]. We have shown an even stronger inverse association using a DXA based estimate of visceral adiposity (abdominal total fat mass) consistent with prior cross-sectional data [15, 20, 42]. Further, we now demonstrate an inverse, longitudinal association between visceral adiposity and SHBG.

Although not directly addressed by the current study, our data are consistent with a considerable body of basic science data that links *de novo* lipogenesis and SHBG production [7, 13, 43, 44]. Prior studies have also suggested that circulating SHBG decreases when fat accumulates in the liver as a result of *de novo* lipogenesis [8, 19, 45]. Taken together these data suggest coupling between the regulation of SHBG and *de novo* lipogenesis. The strong inverse association between serum triglycerides and SHBG levels that we observed accord with the results of previous cross-sectional [11, 18, 21, 31] and limited longitudinal studies [46, 47]. This study is the largest study to date to examine the association of serum triglycerides and SHBG using comprehensive clinical, demographic, anthropometric and bio-psychosocial data simultaneously.

Although SHBG has been shown to be inversely associated with insulin in some cross-sectional and longitudinal studies [7, 11, 13, 15], we found, no association between insulin and SHBG either cross-sectionally or longitudinally. We also found an inverse association between insulin and SHBG among men with normal glucose or those with prediabetes but did not find a significant association between insulin and SHBG among men with T2D (data not shown). Insulin has been reported to directly inhibit hepatic SHBG production [48, 49]. However, there is no direct mechanism by which insulin can regulate transcription of the SHBG gene [23]. Rather, it is likely that the effect is mediated indirectly via inhibition of hepatic *de novo* lipogenesis [23]. This may explain why SHBG tends to be higher in lean individuals with type 1 diabetes, and lower in the presence of obesity with hepatic insulin resistance [3, 45].

Consumption of a diet high in monosaccharides particularly fructose, can reduce serum SHBG levels by about 80% in people without diabetes, and by about 40–50% among those with diabetes [23]. Glucose and fructose reduce SHBG production in HepG2 hepatocarcinoma cells by inducing lipogenesis [44]. We did not examine monosaccharide consumption, but there was no significant association between serum SHBG and serum glucose, as has been shown previously [50, 51]. We did not find any significant association between glucose and SHBG among men categorised as normoglycemic, impaired glucose tolerance and T2D group cross-sectionally but was significant longitudinally. In these data, SHBG levels was significantly low among T2D group (data not shown).

T4 stimulates the production of SHBG in HepG2 hepatocarcinoma cells, indirectly by increasing HNF4-α gene expression and by reducing cellular palmitate levels [48, 52]. T4 has previously been shown to be positively associated with SHBG levels in men with hyperthyroidism and inversely associated with SHBG levels in men who are hypothyroid [53]. Our findings are consistent with these data despite two earlier observations reporting no relationship between T4 and SHBG [15, 54]. As far as we can determine the longitudinal association between T4 and SHBG, that we report, has not previously been demonstrated. Our data also accord with clinical observations of the effect of thyroid hormones on SHBG [10, 11].

SHBG production in HepG2 hepatocarcinoma cells is inhibited by testosterone but the mechanism by which this occurs has not been elucidated [7, 10, 55]. As boys progress through puberty serum SHBG levels decrease as T levels increase [39]. Exogenously administered androgens, even at low doses when taken orally, suppress SHBG [56]. These findings notwithstanding, we found that serum T levels are positively correlated with SHBG cross-sectionally consistent with prior reports [11, 15, 18, 50, 57, 58]. As far as we can determine our data are the first to show a strong positive association between T and SHBG levels longitudinally. Taken together it seems that this reflects the expected steady state relationship between T and SHBG as reflected by the measurement of total T.

The observed inverse association between E2 and SHBG in the cross-sectional data is consistent with the findings of others [12, 15, 16, 20] but stands in contrast to the effect of E2 to increase SHBG in HepG2 hepatocarcinoma cells, an effect most likely dependent on estrogen receptor α (ER-α) mediated upregulation of HNF4-α gene expression [55, 59, 60]. Furthermore, our longitudinal analyses revealed no association between E2 and SHBG. These observations were also confirmed by additional results obtained with a permitted flexible nonlinear associations by restricted cubic spline analyses. The most likely explanation for the difference in cross-sectional *vs*. longitudinal results and findings within the model is that circulating E2 levels are primarily dependent on T, but not age or percentage total fat mass and total T (measured in our study), which in general will be lower when SHBG is lower. The effect of E2 to stimulate SHBG is dependent on a first pass effect through the liver [59]. This applies to both men and women. Oral E2 in women is well described to increase SHBG, whereas topical delivery has a minimal effect [61]. The same is true in men [62]. Moreover, there is a general conclusion from longitudinal studies that serum E_2_ declines when serum T declines in men [63], therefore any effects are likely obscured by the strong positive association between SHBG and total T levels. Support for this explanation comes from a study in which it was shown that with increasing obesity both T and E2 decrease but T does so to a greater extent [64]. Furthermore, we assessed the expression of adipose tissue aromatase in male volunteers to determine the effect of 28 days over feeding a high fat energy dense diet (weight gain) and observed no increase in aromatase per unit of adipose tissue. Accordingly we presume that expanded adipose tissue mass increases biotransformation of T to E accounting for the observed relative preservation of E2 compared with T.

TNF-α at both physiological and supraphysiological concentrations reduces SHBG production in HepG2 cells by down regulating HNF4-α gene expression, an effect mediated via hepatocyte nuclear factor kappa B (NF-κB) [65]. Prior epidemiological studies have reported an inverse association of serum SHBG levels with WBC count and serum fibrinogen but not CRP [66, 67]. We did not find any statistically significant relationships between circulating pro-inflammatory cytokines (TNF-α and IL-6), a marker of macrophage activation (MPO activity), or a marker of vascular endothelial function (eSel) and SHBG. We also did not find any significant association of WBC count with serum SHBG in a subset of the cohort (n = 792) for whom data were available (data not shown).

Taken together these and other data suggest that circulating levels of SHBG are a marker of the presence and severity of hepatic insulin resistance [3], *de novo* lipogenesis [10], and associated conditions including NAFLD [5], type 2 diabetes [10], and cardiovascular disease [7]. From a clinical standpoint the measurement of serum SHBG facilitates the identification of this component of metabolic dysfunction and monitoring of the response to treatment [7, 68]. Interventions that reduce weight, ameliorate insulin resistance, fatty liver and lower serum triglycerides lead to increases in serum SHBG [10]. Conversely worsening of these conditions leads to a decrease in serum SHBG [7, 13]. In patients with type I diabetes, SHBG levels should be normal or raised [69]; a decrease in SHBG level reflects the emergence of hepatic insulin resistance and therefore is a possible indicator of type II diabetes [7, 13]. For instance, SHBG levels were shown to be increased when patients with T2D are treated with rosiglitazone, which reduces insulin resistance by ~30% [45]. A further example is in evaluating the significance of the low testosterone in patients with the metabolic syndrome [13].

The strengths of our study includes the use of a well-characterised, large population based and broadly representative sample of men, with ages ranging from 35 to 80 years, and the simultaneous measurement of numerous potential determinants and confounders. Further, sex steroids were measured, concurrently at each time point, using validated tandem mass spectrometry assays, and body composition was assessed by DXA. Study limitations include the predominant Caucasian population which limits generalisability given known ethnic variations in SHBG [70].

In conclusion, our study has confirmed an age-related rise in circulating SHBG in men, beginning from middle-age. Beyond this gradual increase, reduced SHBG levels largely reflects obesity, particularly men with a substantial or predominant visceral component with associated metabolic consequences. We suggest that variation in SHBG reflects de novo lipogenesis within the liver that occurs in the context of insulin resistance. The observed positive relationship between thyroid hormones and SHBG levels suggests that thyroid hormone may modulate circulating SHBG, either directly or indirectly. High circulating sex steroids are associated with higher SHBG levels reflecting the direct effect of sex steroids.

## Supporting information

S1 Fig Non-linear regression curves of associations between SHBG and potential determinants in community dwelling men. [A] Triglycerides; [B] Glucose; [C] Insulin; [D] ALT; [E] E2; [F] IL-6; [G] TNF-α; [H] eSel, among community dwelling, middle-aged to elderly men. All analyses were adjusted for age, physical activity, smoking status, alcohol consumption, abdominal total fat mass(%), triglycerides, glucose, insulin, alanine transaminases (ALT); free thyroxine (fT4), total testosterone (TT), oestradiol(E2), interleukin 6 (IL-6), tumour necrosis factor alpha(TNF-α), myeloperoxidases (MPO) and e-Selectin (eSel).
(TIFF)

S1 Table Unadjusted, age-adjusted, and multi-adjusted generalized linear regression and lasso regression model to estimate associations between changes in potential determinants (Δ) against change in SHBG (Δ) between visits in community dwelling men (n = 1476). Statistically significant associations (P < 0.05) are shown in bold. Multi-adjusted generalized linear model R2 was 0.240 and lasso regression model R2 was 0.258. Δ = the change in value between baseline and on follow-up over 4.9 years, ALT, alanine transaminases; f T4, free thyroxine; TT, total testosterone; E2,oestradiol; SHBG, sex hormone binding globulin; IL-6,interleukin 6; TNF-α, tumour necrosis factor alpha; MPO, myeloperoxidase; eSel, sE-selectin. a n = 748, b n = 744, c n = 789.
(PDF)

S2 Table Multi-adjusted regression model (via splines) to estimate longitudinal determinants of SHBG in community dwelling men (n = 1476). Data presented are standardised regression coefficients (β) taken from generalized additive models, with cut points determined from corresponding cubic spline analyses and likelihood ratio (S1 Fig). Statistically significant associations (P < 0.05) are shown in bold. ALT, alanine transaminases; E2, oestradiol; IL-6, interleukin 6; TNF-α, tumour necrosis factor alpha; eSel, sE-selectin. a Triglycerides cut-off value was 2 mmol/L; b Glucose cut-off value was 6.0 mmol/L; c Insulin cut-off value was 20.0 μIU/mL; d ALT cut-off value was 45.0 U/L; e E2 cut-off value was 110.0 pmol/L, f IL-6 cut-off value was 3.5 pg/mL; gTNF-α cut-off value was 4.0 pg/mL; h eSel cut-off value was 45.0 ng/mL.
(PDF)

S1 Appendix All relevant data are available in the supporting information files and comprises minimal analytical data set. (SAV)
