# Supplementary figures and images for: Cross-sectional and longitudinal determinants of serum sex hormone binding globulin (SHBG) in a cohort of community-dwelling men
Source: PLoS One. 2018 Jul 11;13(7):e0200078. doi: 10.1371/journal.pone.0200078 (PMC6040731; doi:10.1371/journal.pone.0200078)

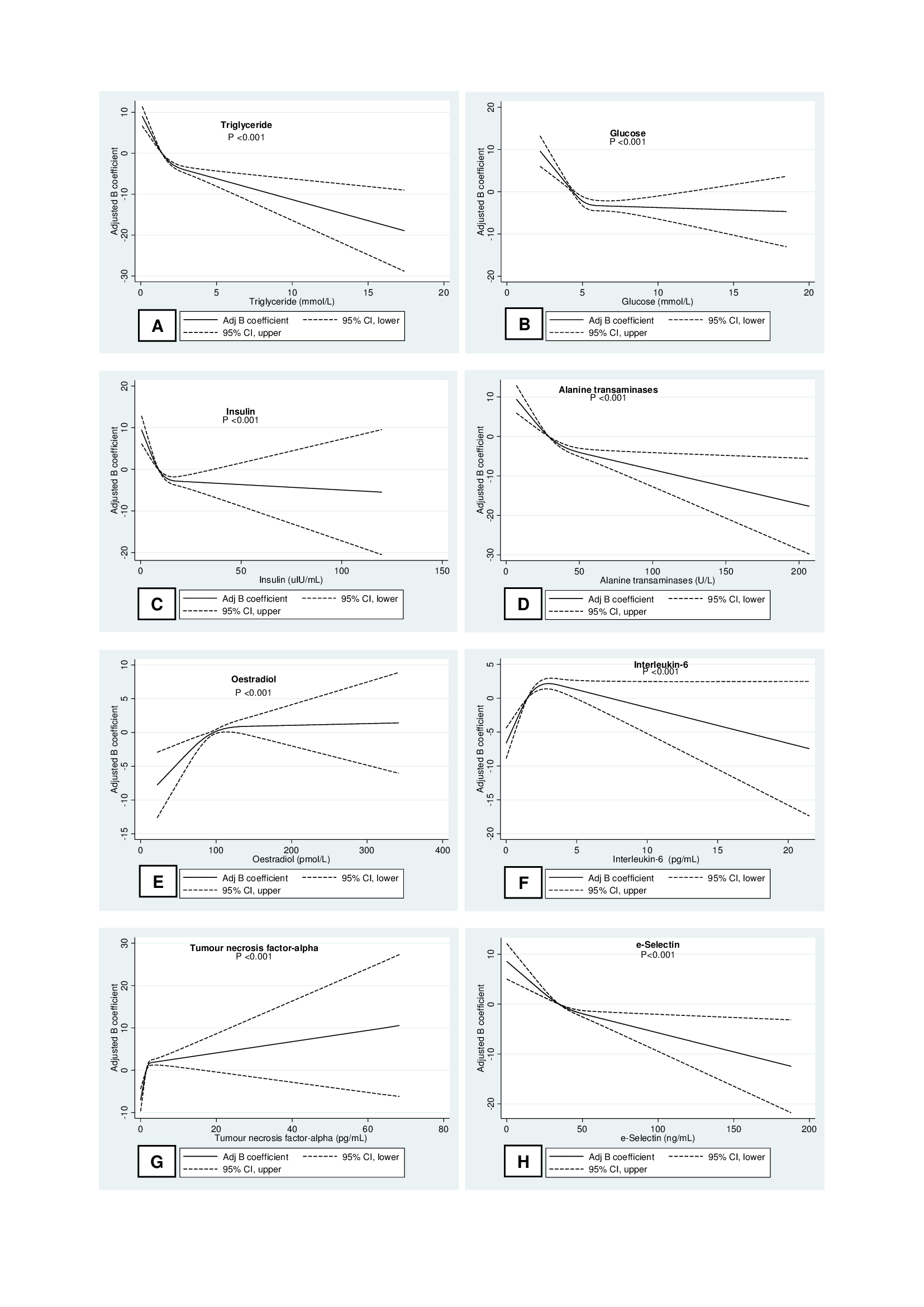

Supplement: S1 Fig — [A] Triglycerides; [B] Glucose; [C] Insulin; [D] ALT; [E] E2; [F] IL-6; [G] TNF-α; [H] eSel, among community dwelling, middle-aged to elderly men. All analyses were adjusted for age, physical activity, smoking status, alcohol consumption, abdominal total fat mass(%), triglycerides, glucose, insulin, alanine transaminases (ALT); free thyroxine (fT4), total testosterone (TT), oestradiol(E2), interleukin 6 (IL-6), tumour necrosis factor alpha(TNF-α), myeloperoxidases (MPO) and e-Selectin (eSel). (TIFF) [file pone.0200078.s001.tiff]
