# Supplementary material for: Cross-sectional and longitudinal determinants of serum sex hormone binding globulin (SHBG) in a cohort of community-dwelling men
Source: PLoS One. 2018 Jul 11;13(7):e0200078. doi: 10.1371/journal.pone.0200078 (PMC6040731; doi:10.1371/journal.pone.0200078)
Supplement: S1 Table — Statistically significant associations (P < 0.05) are shown in bold. Multi-adjusted generalized linear model R2 was 0.240 and lasso regression model R2 was 0.258. Δ = the change in value between baseline and on follow-up over 4.9 years, ALT, alanine transaminases; f T4, free thyroxine; TT, total testosterone; E2,oestradiol; SHBG, sex hormone binding globulin; IL-6,interleukin 6; TNF-α, tumour necrosis factor alpha; MPO, myeloperoxidase; eSel, sE-selectin. a n = 748, b n = 744, c n = 789. (PDF) [file pone.0200078.s002.pdf]

Determinants/factors	ΔSHBG=1.38±9.3								
	Unadjusted model			Age-adjusted		Multi-adjusted (Full model)		LASSO regression	
	Mean difference ± SD	Standardized β	P-value	Standardized β	P-value	Standardized β	P-value	Standardized β	P-value
**Demographic, behavioural & anthropometric factors**									
Δ Age , Years	4.6 ± 0.6	**0.218**	**<0.001**	-	-	0.000	0.999	-	-
Δ Physical activity	148.7 ± 2346.9	0.035	0.450	0.035	0.457	0.032	0.524	-	-
Δ Abdominal total fat mass (%)	-2401.8 ± 894.6	-0.063	0.100	-0.063	0.098	-0.045	0.369	-	-
**Blood chemistry & hormones**									
Δ Triglycerides (mmol/L)	-0.053 ± 1.53	-0.028	0.275	-0.030	0.242	**-0.157**	**0.002**	**-0.165**	**0.001**
Δ Glucose (mmol/L)	0.328 ± 1.257	0.020	0.440	-0.002	0.944	-0.007	0.888	-	-
Δ Insulin (μIU/mL)[a]	-1.2 ± 7.8	-0.058	0.101	-0.060	0.095	0.008	0.876	-	-
Δ ALT activity (U/L)[b]	-2.0 ± 19.7	0.002	0.950	0.003	0.929	0.083	0.106	-	-
Δ fT4 (pmol/L)	1.8 ± 3.3	-0.003	0.919	**0.061**	**0.026**	**0.159**	**0.002**	**0.173**	**0.001**
Δ TT (nmol/L)	-0.631 ± 4.3	**0.333**	**<0.001**	**0.348**	**<0.001**	**0.406**	**<0.001**	**0.403**	**<0.001**
Δ E2 (pmol/L)[c]	-9.3 ± 39.9	**0.074**	**0.033**	0.057	0.107	**-0.114**	**0.035**	**-0.110**	**0.038**
**Inflammatory markers**									
Δ IL-6 (pg/mL)	0.047 ± 1.9	**-0.063**	**0.017**	**-0.074**	**0.004**	**-0.119**	**0.019**	**-0.109**	**0.028**
Δ TNF-α (pg/mL)	0.137 ± 2.9	**-0.079**	**0.003**	**-0.072**	**0.005**	0.041	0.423	-	-
Δ MPO activity (μg/L)	-26.7 ± 329.1	0.004	0.893	-0.005	0.849	0.060	0.249	-	-
Δ eSel (ng/mL)	-0.218 ± 11.4	-0.032	0.277	-0.036	0.164	-0.022	0.676	-	-
