# Supplementary material for: Cross-sectional and longitudinal determinants of serum sex hormone binding globulin (SHBG) in a cohort of community-dwelling men
Source: PLoS One. 2018 Jul 11;13(7):e0200078. doi: 10.1371/journal.pone.0200078 (PMC6040731; doi:10.1371/journal.pone.0200078)
Supplement: S2 Table — Data presented are standardised regression coefficients (β) taken from generalized additive models, with cut points determined from corresponding cubic spline analyses and likelihood ratio (S1 Fig). Statistically significant associations (P < 0.05) are shown in bold. ALT, alanine transaminases; E2, oestradiol; IL-6, interleukin 6; TNF-α, tumour necrosis factor alpha; eSel, sE-selectin. a Triglycerides cut-off value was 2 mmol/L; b Glucose cut-off value was 6.0 mmol/L; c Insulin cut-off value was 20.0 μIU/mL; d ALT cut-off value was 45.0 U/L; e E2 cut-off value was 110.0 pmol/L, f IL-6 cut-off value was 3.5 pg/mL; gTNF-α cut-off value was 4.0 pg/mL; h eSel cut-off value was 45.0 ng/mL. (PDF) [file pone.0200078.s003.pdf]

| Determinants/factors | Multi-adjusted (Full model) | | | | | |
|---|---|---|---|---|---|---|
| | Test below likelihood ratio cut-off | | Test above likelihood ratio cut-off | | Test for overall association (continuous) | |
| | Standardized β | P-value | Standardized β | P-value | Standardized β | P-value |
| **Blood chemistry & hormones** | | | | | | |
| Triglycerides (mmol/L) [a] | **-0.171** | **<0.001** | **-0.143** | **<0.001** | **-0.065** | **0.027** |
| Glucose (mmol/L) [b] | -0.003 | 0.925 | 0.003 | 0.930 | -0.049 | 0.145 |
| Insulin (µIU/mL)[c] | 0.017 | 0.651 | -0.030 | 0.423 | -0.009 | 0.806 |
| ALT activity (U/L)[d] | **0.152** | **<0.001** | **-0.166** | **<0.001** | -0.054 | 0.118 |
| E2 (pmol/L)[e] | **-0.138** | **<0.001** | **0.134** | **<0.001** | -0.023 | 0.490 |
| **Inflammatory markers** | | | | | | |
| IL-6 (pg/mL)[f] | -0.007 | 0.845 | 0.006 | 0.871 | 0.047 | 0.150 |
| TNF-α (pg/mL)[g] | 0.009 | 0.807 | -0.015 | 0.681 | 0.037 | 0.243 |
| eSel (ng/mL)[h] | 0.055 | 0.141 | -0.052 | 0.168 | 0.045 | 0.208 |
